# Clinical impact of first-line therapeutic strategies in BRAF V600E-mutant metastatic colorectal cancer: real-world evidence and prognostic insight

**DOI:** 10.3389/fonc.2025.1608538

**Published:** 2025-07-21

**Authors:** Chia-Chang Yeh, Shih-Wei Chiang, Feng-Fan Chiang

**Affiliations:** ^1^ Division of Colorectal Cancer, Department of Surgery, Taichung Veterans General Hospital, Taichung, Taiwan; ^2^ Department of Nutrition, Chung Shan Medical University, Taichung, Taiwan; ^3^ College of Humanities and Social Sciences, Providence University, Taichung, Taiwan

**Keywords:** BRAF V600E mutation, metastatic colorectal cancer, real-world evidence, first-line therapy, targeted treatment

## Abstract

**Background:**

*BRAF* V600E-mutant metastatic colorectal cancer (mCRC) is associated with poor prognosis and limited response to standard therapies. Recent clinical trials have explored the benefit of targeted therapies, but real-world data remain limited.

**Methods:**

This retrospective study analyzed 36 patients with *BRAF* V600E-mutant mCRC who received first-line treatment between 2018 and 2024 at Taichung Veterans General Hospital. Patients were grouped by initial regimen: chemotherapy alone, chemotherapy plus anti-VEGF (bevacizumab), or chemotherapy combined with *BRAF*-targeted ± MEK inhibitors. Primary endpoints were overall survival (OS) and progression-free survival (PFS); secondary endpoints included objective response rate (ORR) and disease control rate (DCR).

**Results:**

Mean OS and PFS were longest in patients receiving chemotherapy plus anti-VEGF (21.2 and 10.5 months, respectively), compared to chemotherapy alone (OS 14 months, PFS 7.7 months) and anti-BRAF targeted therapy (OS 13.5 months, PFS 6.5 months). The highest ORR (53.8%) and DCR (76.9%) were observed in patients receiving *BRAF*-targeted regimens. Multivariate analysis identified liver metastasis and ECOG ≥2 as poor prognostic factors. Unexpectedly, right-sided tumors were associated with improved survival (HR: 0.20, p = 0.028). Subsequent use of *BRAF*-targeted therapy in some patients may have contributed to extended OS.

**Conclusions:**

In this real-world cohort, chemotherapy combined with anti-VEGF provided the best survival outcomes, while *BRAF*-targeted strategies showed promising response rates. Liver involvement and poor performance status remained negative prognostic indicators. These findings support a personalized treatment approach and highlight the need for continued prospective validation.

## Introduction

Metastatic colorectal cancer (mCRC) remains a major global health challenge, contributing significantly to cancer-related morbidity and mortality worldwide. The *BRAF* V600E mutation, a well-characterized oncogenic driver present in approximately 8–12% of mCRC cases, is associated with a more aggressive disease course, poor prognosis, and resistance to standard treatments (1, 2). Patients harboring this mutation generally exhibit reduced overall survival (OS) and progression-free survival (PFS) compared to their *BRAF* wild-type counterparts, highlighting the need for personalized therapeutic strategies (3).

Standard first-line treatment for mCRC typically includes cytotoxic chemotherapy in combination with monoclonal antibodies targeting vascular endothelial growth factor (VEGF) or epidermal growth factor receptor (EGFR) (4). However, in *BRAF* V600E-mutant mCRC, responses to anti-EGFR agents such as cetuximab and panitumumab are limited due to downstream MAPK pathway activation, which circumvents EGFR blockade (5).

Recent therapeutic advances have introduced combination strategies involving *BRAF* inhibitors (e.g., encorafenib) alongside EGFR inhibitors, and in some cases, MEK inhibitors. These regimens have demonstrated superior efficacy over traditional approaches (6). The Phase III BREAKWATER trial recently evaluated encorafenib plus cetuximab (EC) with mFOLFOX6 compared to standard-of-care (SOC) chemotherapy in first-line *BRAF* V600E-mutant mCRC (7). The trial met its primary endpoint, with EC+mFOLFOX6 achieving a higher objective response rate (ORR) of 60.9% compared to 40.0% with SOC (OR = 2.443, p = 0.0008). Additionally, this regimen demonstrated a longer median duration of response (13.9 vs. 11.1 months) and a favorable interim OS hazard ratio of 0.47. The safety profile was consistent with the known toxicities of each agent.

Despite these advances, the optimal first-line regimen for *BRAF* V600E-mutant mCRC remains should follow the BREAKWATER trial (7). Chemotherapy with anti-VEGF remains a valid standard of care, and encorafenib-cetuximab is also suitable for patients not eligible for chemotherapy. This study aims to evaluate real-world clinical outcomes in 36 patients with *BRAF* V600E-mutant mCRC who received first-line treatment, including analysis of OS, PFS, and response rates. These findings may inform future strategies for managing this high-risk patient population.

## Study designs and methods

This retrospective case series was conducted at Taichung Veterans General Hospital, utilizing data from the institutional mCRC patient registry. The study period spanned from January 1, 2018, to December 31, 2024. *BRAF* V600E mutation status was confirmed via real-time polymerase chain reaction (PCR). Microsatellite instability (MSI) status was assessed using immunohistochemistry (IHC) for mismatch repair proteins.

Eligible patients included those with histologically confirmed mCRC harboring a *BRAF* V600E mutation who had received first-line systemic treatment—either chemotherapy alone or in combination with targeted therapy—and had complete imaging and survival data available. Patients were excluded if they had microsatellite instability-high (MSI-H)/deficient mismatch repair (dMMR) tumors and received immune checkpoint inhibitors, or if their treatment response was unassessable or follow-up incomplete.

Based on the first-line treatment received, patients were categorized into three groups: those who received chemotherapy alone (FOLFOXIRI, FOLFOX, or FOLFIRI), those who received chemotherapy combined with an anti-VEGF agent (bevacizumab), and those who received chemotherapy in combination with a *BRAF* inhibitor plus anti-EGFR therapy, with or without a *MEK* inhibitor.

The primary endpoints were OS, defined as the time from initiation of first-line therapy to death from any cause, and PFS, defined as the time from initiation of therapy to disease progression or death. Secondary endpoints included objective response rate (ORR), defined as the proportion of patients achieving complete or partial response; disease control rate (DCR), which included complete response, partial response, or stable disease.

Survival analyses were performed using Kaplan–Meier methodology, and differences between treatment groups were evaluated using the log-rank test. Prognostic variables—including tumor sidedness, MSI status, and liver metastases—were assessed using a Cox proportional hazards regression model. Categorical variables, such as ORR and DCR across treatment groups, were compared using Fisher’s exact test or the chi-square test, as appropriate.

## Results

### Patient characteristics

A total of 36 patients with *BRAF* V600E-mutant mCRC were included in the study (**Table 1**). The median age was 56.1 years (range, 33–78), and 41.7% (n = 15) were female. Regarding performance status, 44.4% (n = 16) had an Eastern Cooperative Oncology Group (ECOG) score of 0, 47.2% (n = 17) had a score of 1, and 8.3% (n = 3) had a score of 2 or higher. Primary tumors were right-sided in 36.1% (n = 13) and left-sided in 63.9% (n = 23). The majority of patients (86.1%, n = 31) were microsatellite stable (MSS), while 2.8% (n = 1) were MSI-H, and MSI status was unknown in 11.1% (n = 4). Metastases were most frequently observed in two or more organs (33.3%, n = 12), followed by the peritoneum (27.8%, n = 10), liver (22.2%, n = 8), and distal lymph nodes (16.7%, n = 6). No patients presented with lung metastases. The full record of post-first-line treatment regimens, including targeted therapy and chemotherapy sequences, is summarized in **Supplementary Table S1**.

**Table 1:** Patient characteristics.

	*BRAF* V600E mutation patients, n=36
**Age**	56.1 (33- 78)
**Female, (%)**	15 (41.7%)
ECOG, (%)	
0	16 (44.4%)
1	17 (47.2%)
≥ 2	3 (8.3%)
Primary tumor site	
Right-sided	13 (36.1%)
Left-sided	23 (63.9%)
MSI status	
MSI-Stable	31 (86.1%)
MSI-High	1 (2.8%)
Unknown	4 (11.1%)
Metastatic site	
Liver	8 (22.2%)
Lung	0 (0%)
Peritoneum	10 (27.8%)
Distal lymph nodes	6 (16.7%)
≥ 2 organs metastases	12 (33.3%)
First-line regimen	
Chemotherapy only	6 (16.7%)
Chemotherapy + Anti-EGFR	1 (2.8%)
Chemotherapy (Doublet or Triplet)+ Anti-VEGF	15 (41.7%)
Chemotherapy + *BRAF* V600E -targeted therapy	13 (36.1%)
No treatment	1 (2.8%)

(EGFR, Epidermal Growth Factor Receptor; VEGF, ​Vascular Endothelial Growth Factor.).

Bold values indicate statistical significance at p < 0.05.

### Overall response rate and disease control rate

The comparison of treatment response across different first-line regimens showed no statistically significant differences in ORR or DCR among groups (**Table 2**). In the chemotherapy-only group, the ORR was 50% (3 out of 6 patients), compared to 33% (5 out of 15) in the chemotherapy plus anti-VEGF group, and 53.8% (7 out of 13) in the group receiving chemotherapy plus *BRAF*-targeted therapy (p = 0.524). The DCRs were 66.7%, 68.8%, and 76.9% respectively in the same groups (p = 0.895). Although numerical differences were observed, they did not reach statistical significance, suggesting no clear superiority in response or disease control across the treatment regimens in this cohort.

**Table 2:** Objective response rate (ORR) and disease control rate (DCR) among patients with *BRAF* V600E-mutant metastatic colorectal cancer receiving different first-line treatment strategies.

	Chemotherapy-only (n=6)	Chemotherapy plus Anti-VEGF (n=15)	Chemotherapy plus *BRAF* V600Ei-based therapy (n=13)	Chi-square test
ORR	50% (3/6)	33% (5/15)	53.8% (7/13)	0.524
DCR	66.7% (4/6)	68.8%(11/15)	76.9% (10/13)	0.895

(VEGF, Vascular Endothelial Growth Factor; *BRAF* V600Ei-based therapy: *BRAF* V600E inhibitor-based therapy).

### Overall survival and progression free survival

**Figure 1:**
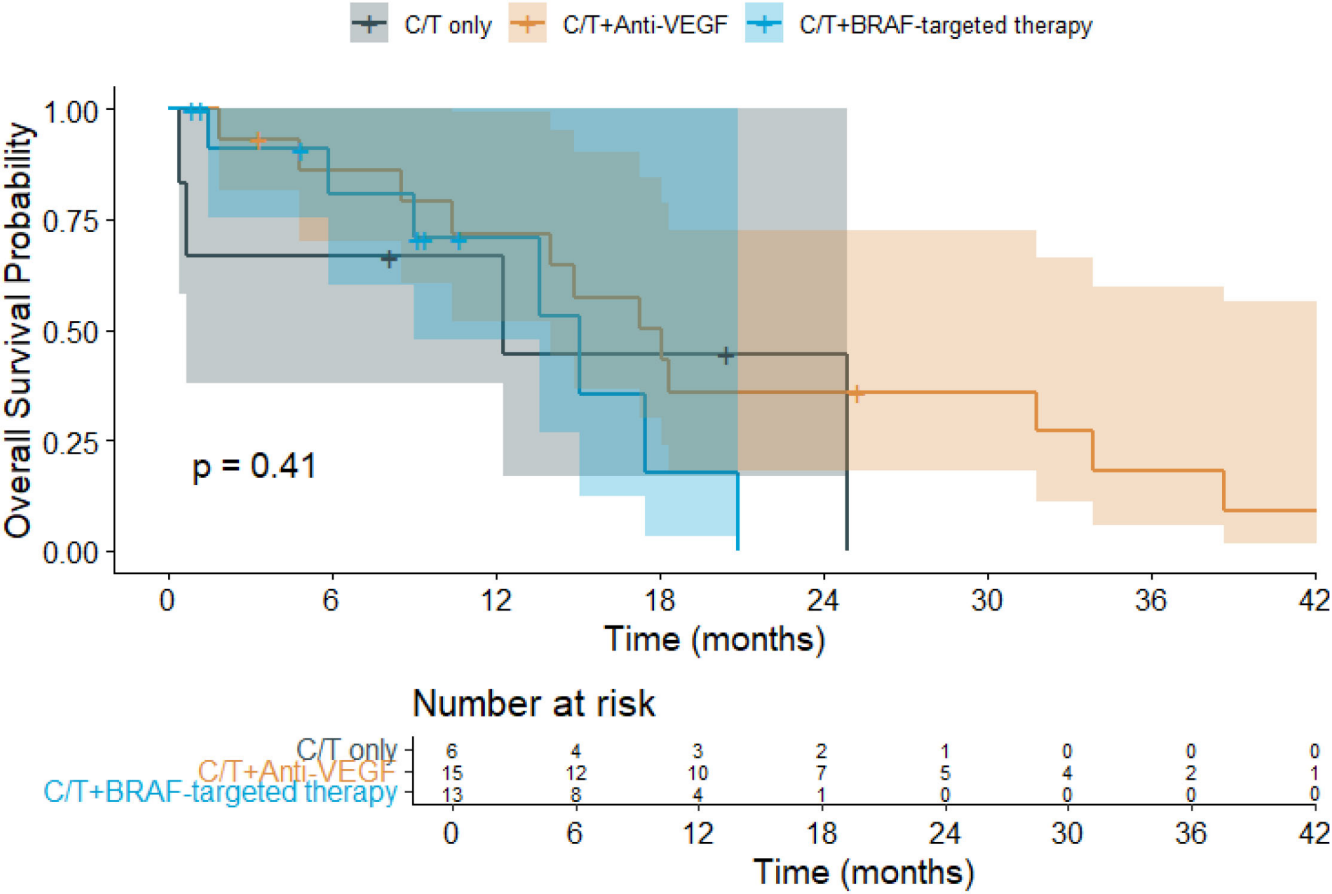
Kaplan–Meier overall survival (OS) curves for patients with *BRAF* V600E-mutant metastatic colorectal cancer (mCRC) receiving different first-line (1L) treatment regimens. (C/T, Chemotherapy; VEGF, ​Vascular Endothelial Growth Factor).

**Figure 2:**
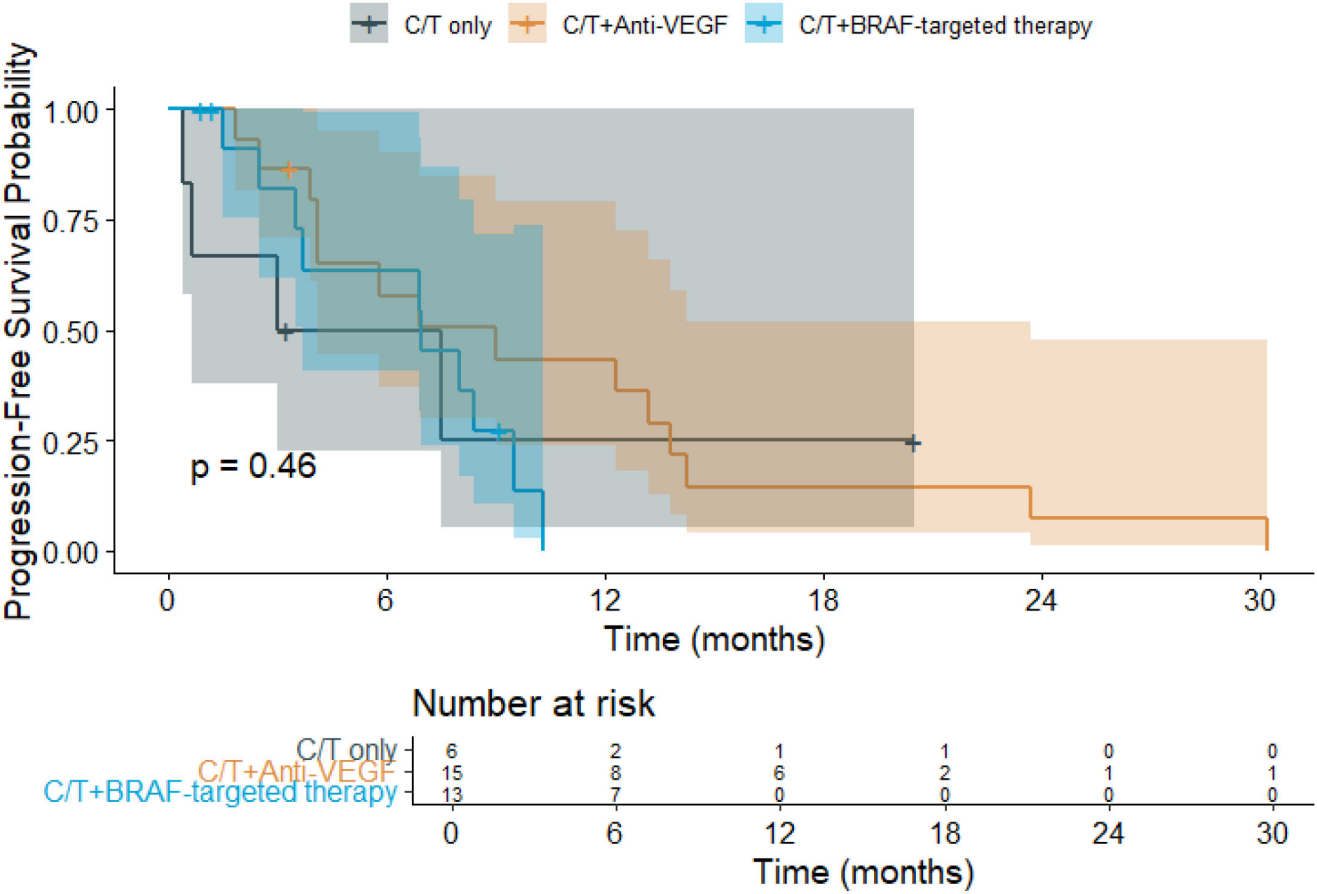
Kaplan–Meier progression-free survival (PFS) curves for patients with *BRAF* V600E-mutant metastatic colorectal cancer (mCRC) receiving different first-line (1L) treatment regimens. (C/T, Chemotherapy; VEGF, Vascular Endothelial Growth Factor).

The median follow-up duration was 13.3 months. Among the treatment groups (**Figure 1**), patients receiving chemotherapy alone had a mean OS of 14.0 months (95% CI: 3.8–24.1), while those treated with chemotherapy plus anti-VEGF experienced the longest mean OS at 21.2 months (95% CI: 13.9–28.5). In contrast, patients receiving chemotherapy combined with *BRAF*-targeted therapy had a mean OS of 13.5 months (95% CI: 9.4–17.6). Regarding PFS (**Figure 2**), the mean duration was 7.7 months (95% CI: 0.9–14.5) for the chemotherapy-only group, 10.5 months (95% CI: 6.1–14.9) for the chemotherapy plus anti-VEGF group, and 6.5 months (95% CI: 4.6–8.3) for the chemotherapy plus *BRAF*-targeted therapy group.

### Sub-group analysis

#### Sideness

**Figure 3:**
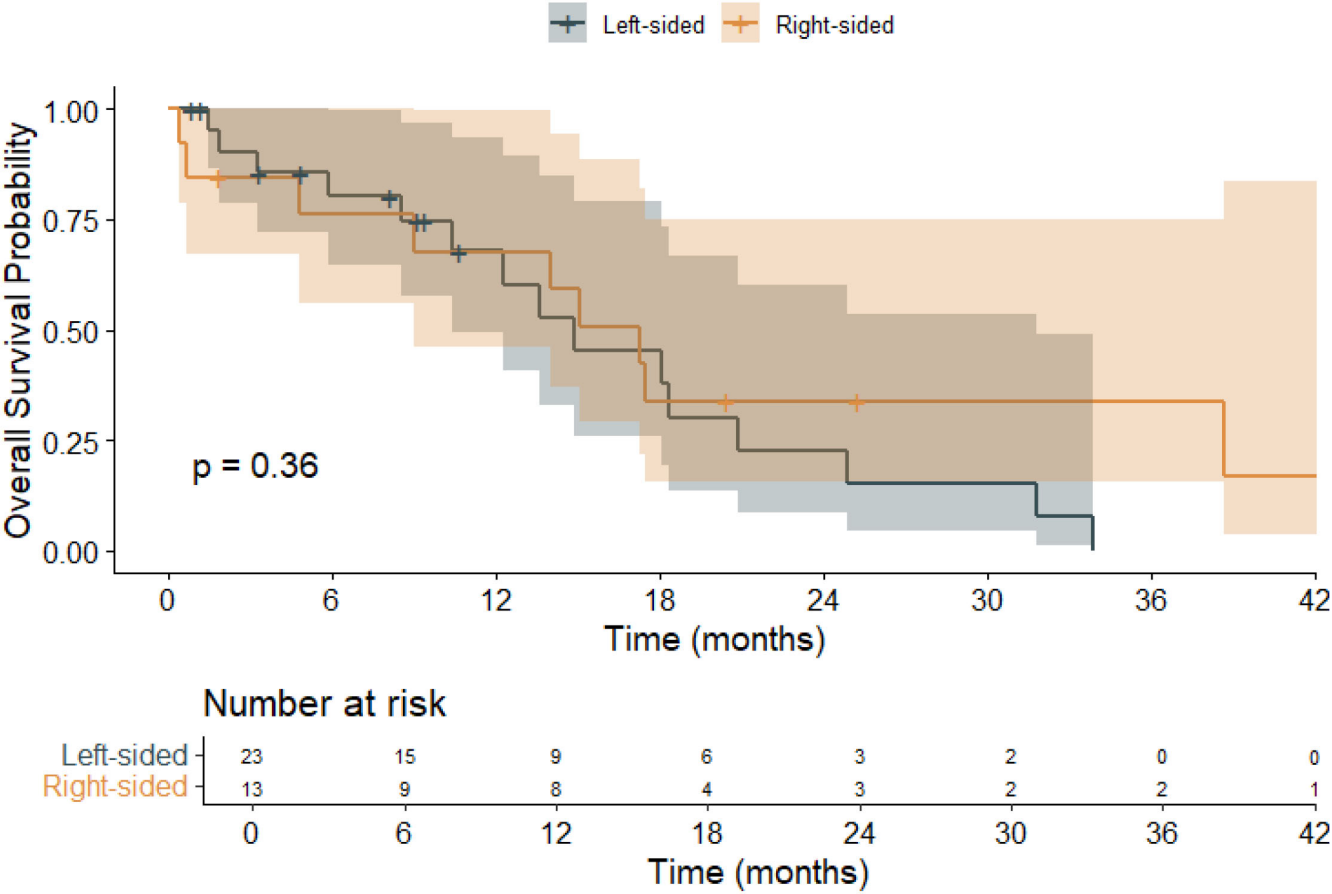
Kaplan–Meier overall survival (OS) curves stratified by primary tumor sidedness in patients with *BRAF* V600E-mutant metastatic colorectal cancer (mCRC).

Kaplan–Meier survival analysis (**Figure 3**) was conducted to evaluate the impact of primary tumor sidedness on OS. Patients with left-sided tumors had a mean OS of 16.0 months (95% CI: 11.2–20.9), while those with right-sided tumors had a longer mean OS of 20.9 months (95% CI: 11.2–30.6). The median OS was 14.9 months (95% CI: 8.3–21.4) for left-sided tumors and 17.3 months (95% CI: 11.8–22.8) for right-sided tumors. The overall mean OS across all patients was 18.0 months (95% CI: 13.2–22.8), with a median OS of 15.1 months (95% CI: 9.95–20.2). These findings indicate a numerically longer OS in patients with right-sided *BRAF* V600E-mutant tumors, although the difference did not reach statistical significance.

#### Liver-involved metastasis

**Figure 4:**
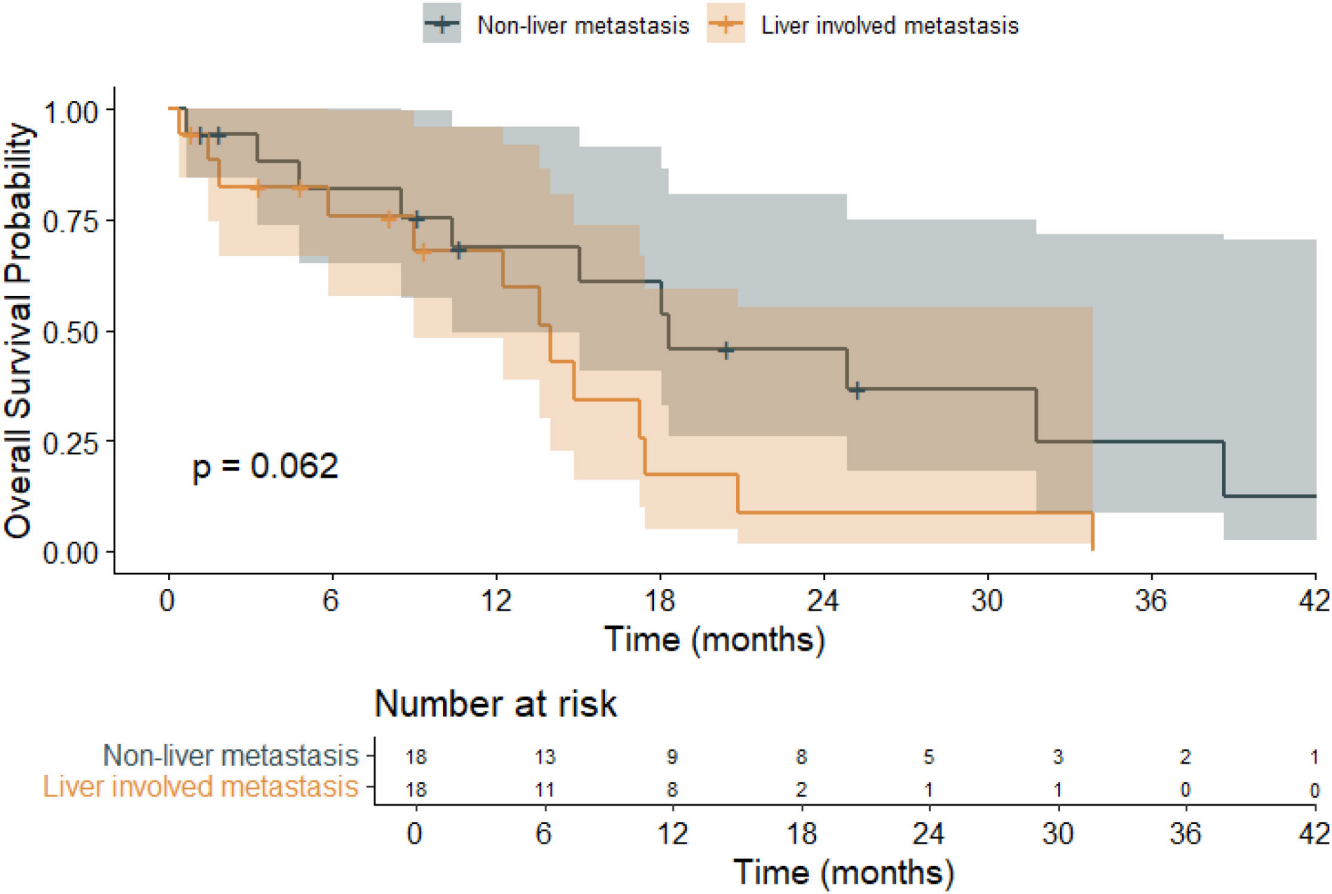
Kaplan–Meier overall survival (OS) curves stratified by liver metastasis or not in patients with *BRAF* V600E-mutant metastatic colorectal cancer (mCRC).

The prognostic impact of liver metastasis on overall survival was also assessed (**Figure 4**). Patients without liver involvement had a mean OS of 22.2 months (95% CI: 14.3–30.0), whereas those with liver metastases had a shorter mean OS of 13.6 months (95% CI: 8.9–18.3). The overall mean OS across all patients was 18.0 months (95% CI: 13.2–22.8). While the log-rank test yielded a p-value of 0.062—falling short of statistical significance—there was a clear trend toward inferior survival in the liver metastasis subgroup, suggesting that hepatic involvement may be a clinically meaningful prognostic factor in *BRAF*-mutant mCRC.

### Multivariate regression analysis

A Cox proportional hazards regression model (**Table 3**) was used to identify independent prognostic factors for overall survival. Liver metastasis was significantly associated with poorer OS (hazard ratio [HR]: 3.80, 95% CI: 1.34–10.74, p = 0.012). Additionally, an ECOG performance status ≥2 was also linked to worse OS outcomes (HR: 2.50, 95% CI: 1.01–6.20, p = 0.047). In contrast, patients with right-sided tumors showed improved OS compared to those with left-sided tumors (HR: 0.20, 95% CI: 0.05–0.84, p = 0.028), a finding that diverges from existing literature and warrants further investigation. Other variables, such as MSI status, sex, age, and the number of metastatic sites, did not show statistically significant associations with survival.

**Table 3:** Multivariate Cox regression analysis of prognostic factors for overall survival in patients with *BRAF* V600E-mutant metastatic colorectal cancer.

Factors	Multivariate		
	HR	95% CI	p-value
1st-line regimen^1^	1.3	0.62- 2.72	0.49
ECOG performance^2^	5.6	2.01- 15.48	<0.001*
Sex	2.5	0.71- 8.58	0.16
Age >65	0.3	0.109- 1.12	0.08
Right-sided primary tumor	0.2	0.04- 0.76	0.02*
≥ 3-lines regimen	0.2	0.04- 0.84	0.03*
Liver-involved metastasis	3.8	1.36- 10.52	0.01*

(HR, Hazard ratios; CI, 95% confidence intervals; *P*, *p*-values).

Multivariate Cox regression analysis for factors associated with overall survival. Statistically significant values are indicated with an asterisk. Reference categories are indicated in footnotes. *Statistically significant (p < 0.05).

¹Reference: chemotherapy only group.

²Reference: ECOG 0–1.

Bold values indicate statistical significance at p < 0.05.

## Discussion

Beyond treatment efficacy, multiple clinical and molecular characteristics have been implicated as prognostic factors in BRAF V600E-mutant metastatic colorectal cancer (mCRC). Notably, poor performance status (ECOG ≥2), liver metastasis, and high tumor burden have been consistently linked to worse survival outcomes (1, 3, 10). In our cohort, ECOG status and liver involvement were independently associated with overall survival, underscoring their prognostic relevance. Primary tumor sidedness has also been proposed as a prognostic indicator in this population, although findings across studies remain inconsistent (13, 14). Moreover, biomarkers such as MSI and concurrent *RAS* mutations have been shown to affect disease biology and prognosis. However, only one MSI-H case was identified in our cohort, suggesting lower prevalence compared to some other real-world and clinical trial populations (4, 5). Further prospective studies incorporating genomic and immune profiling are warranted to better delineate prognostic heterogeneity and optimize treatment stratification.

This real-world analysis of *BRAF* V600E-mutant mCRC patients offers critical insights into the comparative effectiveness of contemporary first-line treatment regimens. Consistent with prior reports, our cohort exhibited a generally poor prognosis, with median OS and PFS across treatment groups falling below those typically observed in *BRAF* wild-type populations. In our study, patients receiving chemotherapy alone had a mean OS of 14 months and PFS of 7.7 months, with an ORR of 50% and DCR of 66.7%. These outcomes are comparable to those reported in the COIN and FOCUS trials, where median OS ranged from 15–16 months and PFS around 7–8 months in chemotherapy-only arms, albeit in largely *BRAF* wild-type populations (8, 9).Given that *BRAF* V600E mutations are typically associated with poor prognosis, the relatively favorable outcomes in our chemotherapy-only subgroup may reflect patient selection. While targeted therapies are increasingly preferred, our findings suggest that conventional chemotherapy remains a reasonable option in selected *BRAF*-mutant patients, particularly when targeted agents are not accessible.

In our cohort, patients receiving chemotherapy plus anti-VEGF (bevacizumab) achieved the longest OS (21.2 months) and PFS (10.5 months), with a DCR of 68.8% and ORR of 33%. Although the response rates were lower than those reported by Loupakis et al. (DCR 88%, ORR 72%), the survival outcomes were comparable. This suggests that bevacizumab-based regimens remain a valid option for selected *BRAF*-mutant mCRC patients, especially when intensive treatment is feasible. While ORR DCR are commonly used indicators of treatment activity, their association with OS in *BRAF* V600E-mutant mCRC is not always linear. In our cohort, the highest ORR (53.8%) and DCR (76.9%) were observed in patients receiving *BRAF*-targeted regimens; however, this group did not achieve the longest OS. Conversely, patients treated with chemotherapy plus anti-VEGF therapy demonstrated lower ORR (33%) but the most favorable OS (21.2 months). This discrepancy may, in part, be explained by treatment sequencing: 7 patients (46.7%) in the anti-VEGF group later crossed over to receive *BRAF*-targeted therapies, potentially contributing to extended survival. A similar trend was observed in the BEACON trial, where pretreated patients with *BRAF* V600E-mutant mCRC receiving encorafenib plus cetuximab experienced improved outcomes in later lines of therapy (6). These findings underscore that in this molecular subtype, durable survival may depend not only on initial response, but also on access to effective sequential treatment. Hence, caution should be exercised when interpreting ORR or DCR as surrogates for long-term benefit in this population.

Patients treated with *BRAF* inhibitor plus anti-EGFR therapy and chemotherapy achieved an ORR of 53.8%, DCR of 76.9%, with a mean OS of 13.5 months and PFS of 6.5 months. Although these outcomes are modest compared to the latest trial data, they remain clinically meaningful. To address treatment heterogeneity within the *BRAF*-targeted therapy group, we compared patients who received doublet versus triplet chemotherapy backbones in combination with BRAF plus EGFR inhibitors (**Supplementary Figures S1**, **S2**). While both groups received dual-targeted regimens, those treated with triplet chemotherapy showed numerically longer OS (16.5 vs. 14.2 months) and PFS (8.3 vs. 7.0 months). Although not statistically significant, this finding suggests a potential benefit of intensified chemotherapy in selected patients receiving targeted therapy.

The BEACON trial demonstrated that encorafenib, binimetinib, and cetuximab significantly improved OS (median 9.3 months) and ORR (26%) over standard chemotherapy in previously treated *BRAF* V600E-mutant mCRC patients (6). More recently, the BREAKWATER phase 3 trial showed that EC plus mFOLFOX6 achieved a superior ORR of 60.9% compared to 40.0% in the standard-of-care arm, with a favorable safety profile and a trend toward improved survival (7). These findings support our real-world observation that multi-agent targeted strategies, when combined with chemotherapy, can yield meaningful clinical benefit in selected patients with *BRAF* V600E-mutant mCRC.

Acknowledge that, likely due to the modest sample size, the study population is enriched for male patients and left-sided tumors. In our multivariate analysis, patients with right-sided primary tumors demonstrated significantly better overall survival compared to those with left-sided tumors (HR: 0.2, p = 0.02). This finding contrasts with previous reports, which generally associate right-sided *BRAF* V600E-mutant mCRC with poorer prognosis.

A retrospective analysis published in Annals of Oncology reported a median OS of 6.6 months in right-sided *BRAF* V600E-mutant mCRC compared to 14.0 months in left-sided tumors (p < 0.01) (10). Similarly, data presented at ASCO showed a median OS of 19.6 months for right-sided tumors and 27.5 months for left-sided tumors (HR: 1.44, p < 0.001) (11). However, Gallois et al. reported no statistically significant OS difference between left- and right-sided tumors treated with encorafenib and cetuximab (12), our multivariate analysis showed significantly better survival in right-sided tumors.

These discrepancies may be attributed to differences in patient characteristics, treatment intensity, and sample size across studies. Our cohort was relatively small, which may have introduced statistical variability. In our multivariate analysis, right-sided tumors were unexpectedly associated with better overall survival (HR: 0.20, p = 0.02), which contradicts most existing literature. This result should be interpreted with caution due to the limited number of patients in this subgroup (n=13), raising the possibility of a type I error. Notably, a pooled analysis by Alig et al. (13). found that left-sided tumors in *BRAF* V600E-mutant mCRC were generally associated with more favorable outcomes and greater benefit from anti-EGFR therapy, whereas right-sided tumors had poorer responses and worse OS, especially in male patients. Recent genomic studies have revealed distinct prognostic subgroups among *BRAF*-mutant colorectal cancers, influenced by microsatellite status, tumor location, and immune landscape (14). Differences in immune gene expression between right- and left-sided *BRAF*-mutant tumors may contribute to heterogeneous treatment responses and outcomes (15). Additionally, the biological behavior and treatment responsiveness of left- versus right-sided tumors differ and may interact with *BRAF*-driven oncogenic pathways. Further large-scale prospective studies are warranted to validate our findings and clarify the prognostic implications of tumor sidedness in *BRAF* V600E-mutant mCRC.

Liver metastasis was identified as an independent poor prognostic factor for both overall survival (OS) and progression-free survival (PFS) in patients with *BRAF* V600E-mutated mCRC, with a hazard ratio (HR) of 2.71 for OS and 2.12 for PFS in the multivariate analysis. These findings are consistent with results from the CONFIDENCE study, where liver metastases were also associated with worse outcomes (PFS HR = 2.037; 95% CI: 1.06–3.91, P = 0.032) (16). Similarly, Meng et al. reported a significant impact of liver metastasis on OS (HR = 2.399; 95% CI: 1.242–4.635, P = 0.009) in a Chinese real-world cohort of *BRAF*-mutant mCRC (17). Recent efforts to refine prognostic stratification in BRAF V600E-mutant mCRC have led to the development of composite risk models. The BRAF BeCool study by Loupakis et al. proposed a validated prognostic classifier incorporating ECOG performance status, tumor burden, and metastatic sites, which successfully stratified patients into low-, intermediate-, and high-risk groups with significantly different survival outcomes (18). Our real-world data similarly identified ECOG ≥2 and liver involvement as independent predictors of poor overall survival, in line with the *BRAF* BeCool model, although external validation in broader cohorts remains warranted. The plasmatic BRAF V600E allele fraction has been identified as a prognostic biomarker demonstrated that higher baseline ctDNA levels were associated with worse survival in patients receiving *BRAF*-targeted therapy (19).

Access to *BRAF*-targeted therapy may have influenced treatment patterns in our real-world cohort. In Taiwan, at the time of this study, anti-*BRAF* agents such as encorafenib were not yet reimbursed or formally approved for first-line use in metastatic colorectal cancer. As a result, some clinicians opted for off-label use of *BRAF*-targeted therapies in selected patients, particularly in later-line settings. These variations in drug accessibility and regulatory constraints likely contributed to heterogeneity in treatment sequencing and may have affected survival outcomes. Similar real-world observations from European cohorts suggest that sequential *BRAF*-targeted therapies may improve survival beyond first-line treatment (18). In regions with limited reimbursement for *BRAF*-targeted agents, off-label use has been reported in real-world settings, reflecting a need for flexible treatment approaches (20).

In addition, heterogeneity in the use of anti-MEK agents and evolving treatment paradigms during the study period complicate cross-group comparisons. Long-term follow-up from real-world cohorts also supports the durable efficacy of *BRAF*/MEK-targeted strategies outside of clinical trials (21).

Several limitations must be acknowledged. The retrospective design and limited sample size may have hindered the detection of statistically significant differences and introduced selection bias. One notable limitation of this study is the relatively small sample size (n = 36), which was further divided into three treatment groups. This stratification limited the statistical power of intergroup comparisons. A *post-hoc* power analysis was conducted using observed mean overall survival and estimated variance across the three groups. The calculated effect size (f = 0.33) corresponded to a statistical power of only 35.7% at a significance level of 0.05, which falls considerably short of the conventional 80% threshold. This suggests a substantial risk of Type II error, meaning that potentially meaningful differences may not have reached statistical significance. To achieve adequate power for detecting differences of this magnitude, a minimum sample size of approximately 81 patients would be required. These findings underscore the need for future multi-institutional studies with larger cohorts to validate our observations and provide more definitive guidance for clinical decision-making in this high-risk population.

## Conclusions

This real-world study highlights the heterogeneity in clinical outcomes among patients with *BRAF* V600E-mutant mCRC receiving first-line treatment. Chemotherapy combined with anti-VEGF therapy yielded the most favorable survival, while *BRAF*-targeted strategies showed encouraging response rates, particularly when integrated with subsequent therapy lines. Liver metastasis and poor performance status were identified as adverse prognostic factors. These findings support the continued refinement of personalized treatment strategies and reinforce the need for prospective trials to optimize care in this challenging patient population.

## Data Availability

The raw data supporting the conclusions of this article will be made available by the authors, without undue reservation.
