# Supplementary material for: Clinical impact of first-line therapeutic strategies in BRAF V600E-mutant metastatic colorectal cancer: real-world evidence and prognostic insight
Source: Front Oncol. 2025 Jul 21;15:1608538. doi: 10.3389/fonc.2025.1608538 (PMC12318728; doi:10.3389/fonc.2025.1608538)

**Supplement material**

**Table S1.** Subsequent treatment regimens received by each patient following first-line therapy

|  | Chemotherapy | | | | | | | | | | | | | | | **OS** | **PFS** |
| --- | --- | --- | --- | --- | --- | --- | --- | --- | --- | --- | --- | --- | --- | --- | --- | --- | --- |
| No.1 | FOLFIRI | | | | | | | | | | | | | | | 0.4 | 0.4 |
| No.2 | XELOX | | | | | | | XELOXIRI | | | | | | | | 24.9 | 7.5 |
| No.3 | FOLFOX | | | | | | | | | | | | | | | 0.7 | 0.7 |
| No.4 | Ufur | | | | | | | | | | | | | | | 12.3 | 3.0 |
| No.5 | FOLFOXIRI | | | | | | | | | | | | | | | 20.5 | 20.5 |
| No.6 | FOLFOX | | | | | | | | | | | | | | | 8.1 | 3.3 |
|  | Chemotherapy plus Anti-VEGF | | | | | | | | | | | | | | |  |  |
| No.7 | FOLFIRI + **Bevacizumab** | FOLFIRI + **Cetuximab** | | | FOLFOXIRI + **Cetuximab** + **Vemurafenib** | | | **Regorafenib** | | | | **Trifluridine/Tipiracil** | | | **Dagrafenib** + **Anti-MEK inhibitor** | 31.8 | 9.0 |
| No.8 | FOLFOXIRI + **Bevacizumab** | | | | | | | | | | | | | | | 17.3 | 3.9 |
| No.9 | FOLFIRI + **Bevacizumab** | | | | | | | | FOLFIRI + **Cetuximab** + **Vemurafenib** | | | | | | | 38.7 | 23.7 |
| No.10 | FOLFOXIRI + **Bevacizumab** | | | FOLFIRI + **Cetuximab** | | | | | | | **Regorafenib** + Irinotecan | | | | | 33.9 | 12.3 |
| No.11 | FOLFIRI + **Bevacizumab** | | | FOLFOX | | | | | | | Ufur | | | | | 18.3 | 13.2 |
| No.12 | FOLFOXIRI + **Bevacizumab** | | | | | | | | FOLFOX + **Bevacizumab** | | | | | | | 8.5 | 6.9 |
| No.13 | FOLFOXIRI + **Bevacizumab** | | | | | | | | Ufur | | | | | | | 45.5 | 30.2 |
| No.14 | FOLFOX + **Bevacizumab** | | | FOLFOXIRI + **Bevacizumab** | | | | | | | Dagrafenib + Trametinib + **Cetuximab** | | | | | 10.4 | 5.8 |
| No.15 | FOLFOXIRI + **Bevacizumab** | | Irinotecan + **Cetuximab** + **Vemurafenib** | | | | **Regorafenib** | | | **Trifluridine/Tipiracil** | | | | XELIRI | | 18.1 | 2.5 |
| No.16 | FOLFOXIRI + **Bevacizumab** | | | | | | | | FOLFIRI + **Cetuximab** + **Vemurafenib** | | | | | | | 14.0 | 4.1 |
| No.17 | FOLFOXIRI + **Bevacizumab** | | | | | | | | | | | | | | | 4.8 | 4.1 |
| No.18 | FOLFOXIRI + **Bevacizumab** | | | | | | | | **Encorafenib** + **Cetuximab** + Ufur | | | | | | | 14.9 | 14.3 |
| No.19 | FOLFIRI + **Bevacizumab** | | | | | | | | | | | | | | | 1.9 | 1.9 |
| No.20 | XELIRI + **Bevacizumab** | | | | | | | | | | | | | | | 3.4 | 3.4 |
| No.21 | FOLFOXIRI + **Bevacizumab** | | | | | | | | **Encorafenib** + **Cetuximab**+ 5-Fu | | | | | | | 25.3 | 13.8 |
|  | Chemotherapy plus *BRAF* V600E -targeted therapy | | | | | | | | | | | | | | |  |  |
| No.22 | Ufur + **Panitumumab** + **Vemurafenib** | | | | | | | | FOLFIRI + **Bevacizumab** | | | | | | | 15.1 | 8.4 |
| No.23 | **Panitumumab** + **Dagrafenib** | | | | | | | | | | | | | | | 1.5 | 1.5 |
| No.24 | FOLFOX + **Panitumumab** + **Vemurafenib** | | | | | | | | XELIRI + **Bevacizumab** | | | | | | | 9.0 | 3.7 |
| No.25 | FOLFOX + **Panitumumab** + **Vemurafenib** | | | | | | | | XELIRI + **Bevacizumab** | | | | | | | 17.5 | 10.3 |
| No.26 | FOLFOXIRI + **Panitumumab** + **Vemurafenib** | | | | | **Panitumumab** + **Vemurafenib** | | | | | | | **Regorafenib** | | | 20.9 | 6.9 |
| No.27 | FOLFOXIRI + **Vectibix** + **Vemurafenib** | | | | | Ufur + **Panitumumab** + **Vemurafenib** | | | | | | | FOLFOXIRI + **Bevacizumab** | | | 13.6 | 9.5 |
| No.28 | FOLFOX + **Encorafenib** + **Cetuximab** | | | | | | | | | | | | | | | 9.1 | 9.1 |
| No.29 | FOLFIRI + **Encorafenib** + **Panitumumab** | | | | | | | | | | | | | | | 10.7 | 8.0 |
| No.30 | FOLFOX + **Encorafenib** + **Cetuximab** | | | | | | | | **Trifluridine/Tipiracil** +**Bevacizumab** | | | | | | | 5.9 | 2.5 |
| No.31 | FOLFIRI + **Encorafenib** + **Panitumumab** | | | | | FOLFOX + **Bevacizumab** | | | | | | | **Bevacizumab** + **Trifluridine/Tipiracil** | | | 9.4 | 7.0 |
| No.32 | **Encorafenib** + **Cetuximab** | | | | | | | | FOLFIXIRI + **Bevacizumab** | | | | | | | 4.9 | 3.5 |
| No.33 | FOLFOX + **Encorafenib** + **Cetuximab** | | | | | | | | | | | | | | | 0.9 | 0.9 |
| No.34 | FOLFIRI + **Encorafenib** + **Panitumumab** | | | | | | | | | | | | | | | 1.2 | 1.2 |
|  | Other treatments | | | | | | | | | | | | | | |  |  |
| No.35 | **No treatment** | | | | | | | | | | | | | | | 1.9 | 1.9 |
| No.36 | FOLFIRI + **Cetuximab** | | | | | | | | | | | | | | | 3.3 | 2.8 |

OS (overall survival) and PFS (progression-free survival) durations are listed individually. Abbreviations: FOLFOX (leucovorin, 5-FU, oxaliplatin); FOLFIRI (leucovorin, 5-FU, irinotecan); FOLFOXIRI (leucovorin, 5-FU, oxaliplatin, irinotecan); XELOX (capecitabine, oxaliplatin); XELIRI (capecitabine, irinotecan); Ufur (tegafur + uracil); VEGF (vascular endothelial growth factor).

| \| First line treatment \| **Number of patients** \| **OS** \| **PFS** \| \| --- \| --- \| --- \| --- \| \| Chemotherapy \| 6 \| 11.1 \| 5.9 \| \| Chemotherapy plus Anti-VEGF \| 15 \| 19.1 \| 9.9 \| \| Chemotherapy plus *BRAF* V600E -targeted therapy \| 13 \| 9.2 \| 5.6 \| \| Other treatment \| 2 \| 2.6 \| 2.3 \| |
| --- | --- | --- | --- | --- | --- | --- | --- | --- | --- | --- | --- | --- | --- | --- | --- | --- | --- | --- | --- | --- |

**Figure S1.** Kaplan–Meier curves for overall survival (OS) in patients receiving *BRAF*-targeted therapy stratified by chemotherapy intensity (doublet vs. triplet).


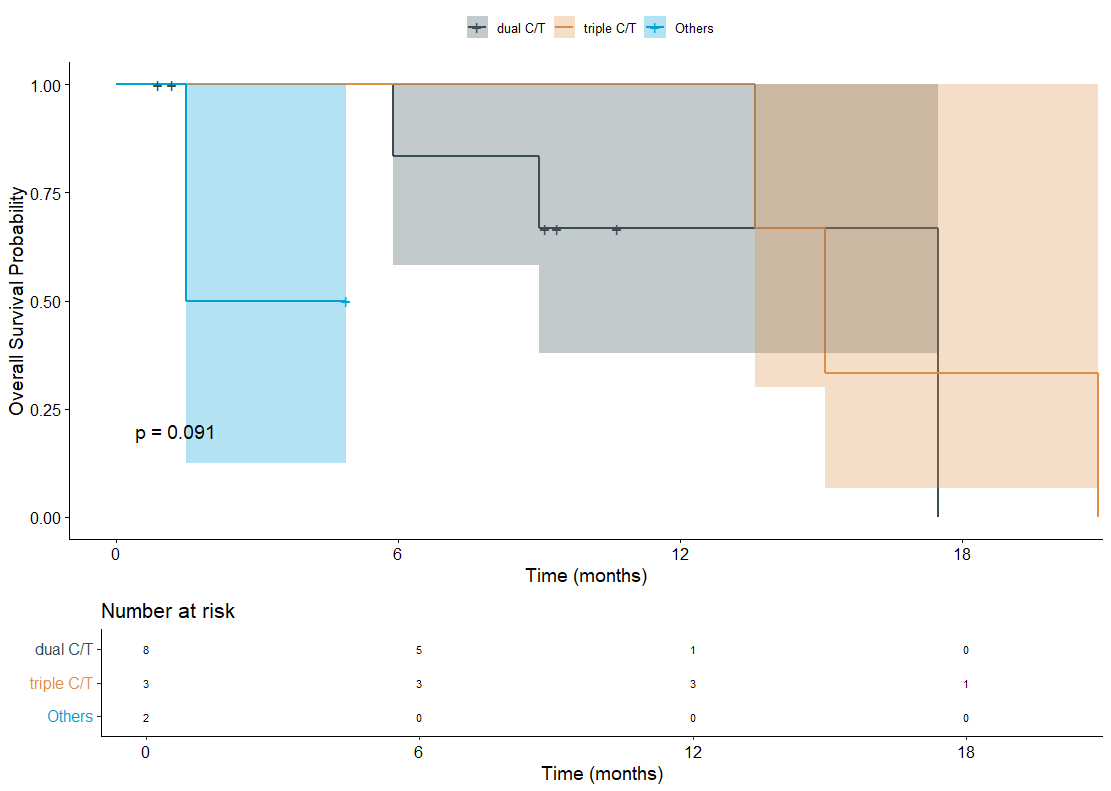


**Figure S2.** Kaplan–Meier curves for progression-free survival (PFS) in patients receiving *BRAF*-targeted therapy, comparing doublet and triplet chemotherapy regimens.


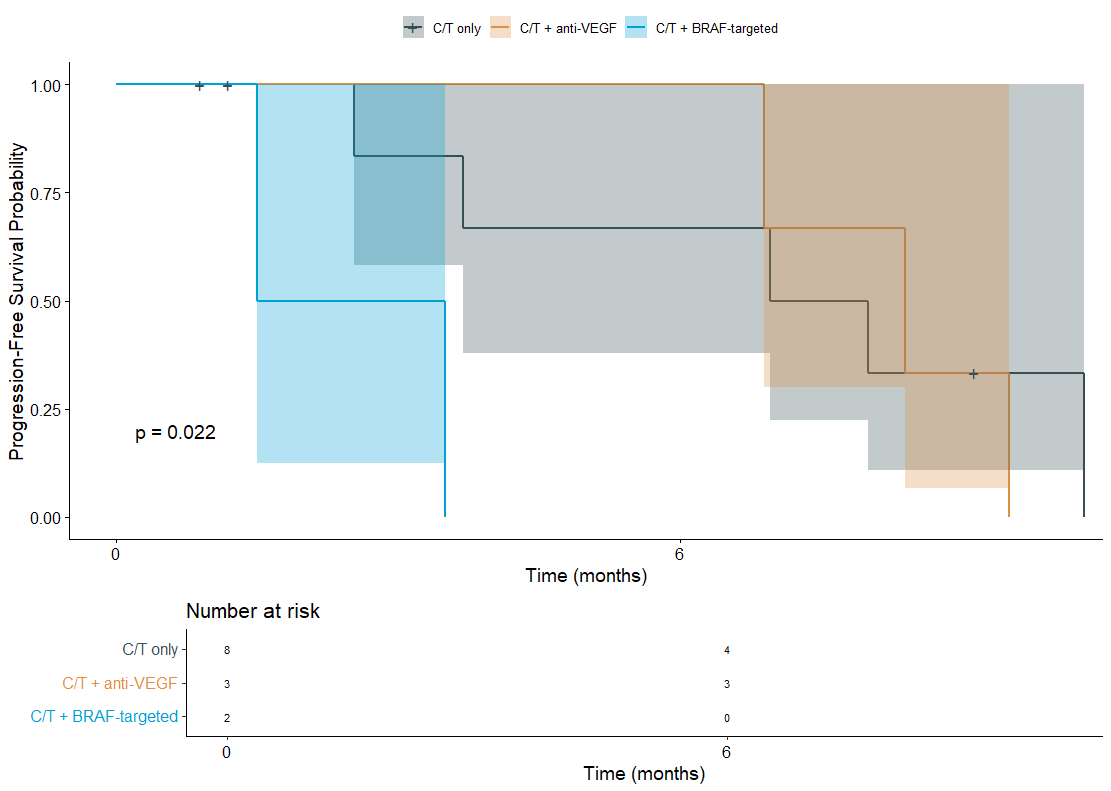

Supplement: Supplementary file 1 [file DataSheet1.docx]
